# Adverse Events for Monoclonal Antibodies in Patients with Allergic Rhinitis: A Systematic Review and Meta-Analysis of Randomized Clinical Trials

**DOI:** 10.3390/jcm12082848

**Published:** 2023-04-13

**Authors:** Yuxi Lin, Weiqing Wang, Zhenzhen Zhu, Surita Aodeng, Lei Wang, Yuzhuo Liu, Jingjing Li, Yang Zha, Xiaowei Wang, Wei Lv

**Affiliations:** 1 4+4 Medical Doctor Program, Chinese Academy of Medical Sciences and Peking Union Medical College, Beijing 100006, China; 2 Department of Otolaryngology, Peking Union Medical College Hospital, Chinese Academy of Medical Science and Peking Union Medical College, Beijing 100006, China

**Keywords:** allergic rhinitis, monoclonal antibodies, biologics, biological therapies, adverse events, safety, meta-analysis

## Abstract

(1) Background: Allergic rhinitis (AR) is a common disease in otolaryngology and novel biological therapies are required for clinical needs. To assess the tolerability of monoclonal antibodies, justifying their clinical applications, we presented a comprehensive safety profile of biologics in AR; (2) Methods: A systematic literature search was conducted following PRISMA guidelines for randomized clinical trials comparing monoclonal antibodies and placebo in AR. PubMed, Web of Science, Medline, and Cochrane were searched up until 9 January 2023. Among 3590 records in total, 12 studies with more than 2600 patients were included. Quality was assessed for all studies using Cochrane risk-of-bias tool for randomized trials, and subgrouped meta-analysis was performed; (3) Results: We accomplished an up-to-date literature overview and analysis on adverse events of monoclonal antibodies in AR. Total, common, severe, discontinuation-causing, and serious adverse events failed to reach statistical significance. Country was an essential factor for heterogeneity, and urticaria was the adverse event at highest risk (RR 2.81, 95% CI 0.79–9.95); (4) Conclusions: Monoclonal antibodies are considered well tolerated and relatively safe in patients with AR. The regions of patients and hypersensitive adverse reactions such as urticaria require a special caution in biological treatments in AR.

## 1. Introduction

Allergic rhinitis (AR) is one of the most common diseases in otolaryngology, affecting about 10–40% population worldwide [1], and its prevalence rate has increased progressively, especially in developed countries [2]. Classic symptoms of AR include nasal itching, sneezing, rhinorrhea, and nasal congestion. Additionally, ocular symptoms such as allergic rhinoconjunctivitis are common, causing itching and redness of the eyes and tearing, and other symptoms are itching of the palate, postnasal drip, and cough [3]. AR was traditionally subdivided into seasonal, perennial, and occupational rhinitis. Seasonal allergic rhinitis (SAR) is frequently induced by outdoor allergens such as pollens or molds, while perennial allergic rhinitis (PAR) is most often induced by indoor allergens such as dust mites, animal dander, molds, and cockroaches. Current classification is based on the severity of symptoms, including sleep disturbance, impairment of daily activities, leisure and/or sport, impairment of school or work, and troublesome symptoms. “Mild” indicates no mentioned symptoms, and “moderate-severe” means at least one symptom is present [3]. There are various traditional treatments of AR, including education, allergen avoidance, pharmacotherapy such as antihistamines and corticosteroids, and allergen-specific immunotherapy (AIT) [4,5]. Given traditional treatment regimens are not always efficacious, there is certainly a role for newer therapies, such as monoclonal antibodies (mAbs) [6]. As the progress of researches on the immunopathogenic mechanisms of AR, mAbs blocking essential disease-causing factors are proven to have promising therapeutic effects [7,8,9,10,11,12]. However, the overall safety of mAbs is still in question, with a poorly understanding of the underlying mechanisms of many mAb-related adverse reactions [13]. According to the FDA label of omalizumab (XOLAIR), common side effects include injection site reaction (45%), viral infections (23%), upper respiratory tract infection (20%), sinusitis (16%), headache (15%), and pharyngitis (11%); anaphylaxis and malignancies are the most serious adverse reactions occurring in clinical trials [14]. Besides, injection site reactions are also major adverse events in many other biological products. For example, a meta-analysis demonstrated that dupilumab increased the risk of injection site reactions in patients with allergic diseases [15]. Safety assessment on novel biological therapies is a crucial aspect and one of the fundamental criteria, as much as efficacy, for the justification of their clinical applications [16]. Therefore, a comprehensive safety profile of biologics in AR is certainly needed before widespread use.

In this systematic review, we aimed to exhaustively search and summarize studies investigating monoclonal antibody treatments and their adverse events in AR patients. In the following step, meta-analysis was conducted to statistically analyze the risk of total, common, severe, withdrawal-causing, and serious adverse events and assess the safety of mAb treatments in patients with AR from different aspects. It allowed us to summarize the current knowledge of adverse events in mAb RCTs and perform a comprehensive safety assessment to specify tolerability information on monoclonal antibody therapies in AR for novel clinical drug selections in the future.

## 2. Methods

### 2.1. Search Strategy

We searched PubMed, Web of Science, Medline and the Cochrane Central Register of Controlled Trials databases from inception to 9 January 2023 without language restrictions. Combinations of AR-related terms (“allergic rhinitis”, “rhinitis”, “rhino conjunctivitis”, “nasal allergy”, or “hay fever”), mAb-related terms (“monoclonal antibodies”, “humanized”, “anti-IgE”, “omalizumab”, “anti-IL4”, “anti-IL13”, “anti-IL4Ra”, or “dupilumab”) and therapy-related terms (“therapy”, “treatment”, or “management”) were used when screening titles/abstracts/keywords of articles. We also manually searched reference lists and similar articles for additional relevant studies. Randomized controlled trials (RCTs) reporting the efficacy and safety of mAbs for the treatment of allergic rhinitis against placebo were retrieved for a full-text review and assessed for eligibility. For missing, unclear, or incomplete results, we contacted researchers for clarification before exclusion. Studies that met the inclusion criteria were included for further analysis.

### 2.2. Selection Criteria

To reduce selection bias, two reviewers independently assessed each study and disagreements were resolved by consensus with a third reviewer, if necessary. The selection criteria were set prior to the literature search process. Studies that met the following criteria were eligible for inclusion: (1) the study population comprised allergic rhinitis patients of any age groups, confirmed with a physician diagnosis and evidence of clinically relevant allergic sensitization; (2) RCTs comparing the use of monoclonal antibodies therapy with placebo; (3) safety assessment was accomplished by reporting adverse events.

Studies were excluded if: (1) allergic rhinitis was treated as a clinical manifestation or optional comorbid of other diseases; (2) the study design didn’t follow RCTs; (3) patients were treated with any mAbs in the past 12 months before studies started; (4) the control group received treatment other than placebo; (5) the treatment group received mAbs along with any other treatments instead of mAbs alone.

### 2.3. Data Extraction and Analysis

Two investigators independently read through the included studies and recorded basic information such as first author and year of publishment. We also collected information on characteristics of the included RCTs, including countries where trials were conducted, sample size, monoclonal antibody given to the treatment group, intervention scheme, and follow-up time. Moreover, patients’ baseline demographics in the included studies were collected, including age (range and mean), sex (male/female), race (White/Black/Asian/others), weight (range and mean), type of AR (SAR/PAR), serum IgE (mean), and history of asthma and atopic dermatitis. Most importantly, for all included RCTs, we collected safety information about the frequency and detailed clinical manifestations of adverse events. 

Meta-analysis was conducted through RStudio using Mantel-Haenszel method. Heterogeneity was evaluated according to heterogeneity test and I^2^ statistic. A *p*-value < 0.1 was considered as a significant heterogeneity. Meanwhile, I^2^ of 25%, 50%, and 75% represented a low, medium, and high heterogeneity, respectively. For I^2^ < 50%, a fixed-effects model was used for meta-analysis. However, if I^2^ > 50%, indicative of a high heterogeneity, a random-effects model should be used to maintain conservative. Lastly, we used RStudio to conduct subgroup analysis and present forest plots.

### 2.4. Quality Assessment

Two investigators independently conducted the quality assessment for each included study using Cochrane risk-of-bias tool for randomized trials (RoB 2). We assessed 6 domains representing the selection, performance, detection, attrition, and reporting bias, and evaluated the risk as 3 levels: low, unclear, and high [17]. The study was identified to be at low risk only if all 6 domains were at low risk. If at least 1 domain was at high risk, the overall risk level was high. If none of the domain was at high risk, but at least 1 domain had unclear risk, we considered the study as unclear risk. Furthermore, publication bias was assessed using funnel plots.

## 3. Results

### 3.1. Study Selection, Characteristics, and Quality

**Figure 1:**
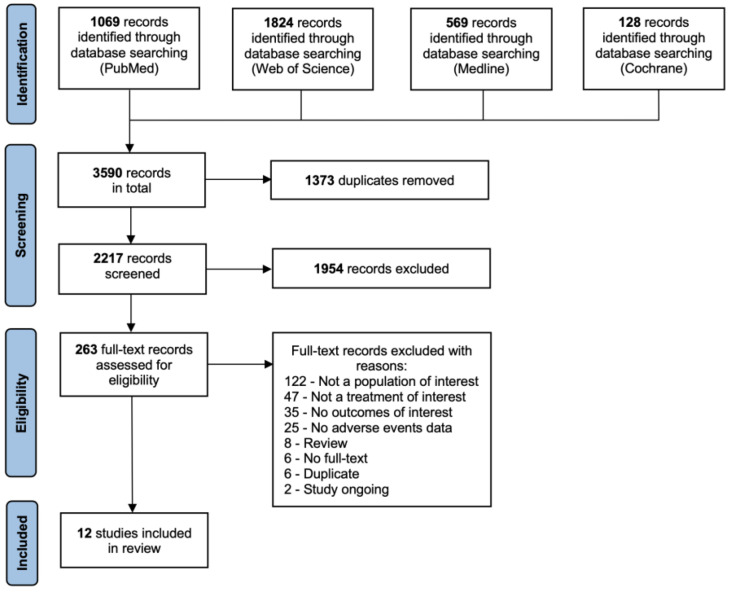
PRISMA flow chart for the process of study selection and inclusion [18].

The systematic search resulted in 3590 records in total from 4 sources. After 1373 duplicates were identified using Mendeley Desktop (version 1.19.8) and removed manually, 2217 records were roughly screened using title and abstract for clinal trials meeting the inclusion criteria. Out of these, 263 full-text records were assessed for eligibility, and 12 studies were included in the following systematic review. The process of literature search and study selection until the final decision on included studies was presented in the PRISMA flow chart in Figure 1.

Among the included studies, 11 of them were published in academic journals from 1997 to 2022, and 1 presented data on governmental website for clinical trials (Identifier: NCT04709575) with no official publications. There were 6 trials conducted in only one country: 3 in the USA, 1 in Japan, 1 in Canada, and 1 in Belgium. The other 6 trials were multinational, including countries in North America, South America, Europe, Asia-Pacific, and Africa. The total sample size of included studies varied between 36 and 536 patients, while the experimental sample size varied from 24 to 400. For the included 12 trials, 7 of them investigated the safety of anti-IgE monoclonal antibodies, 2 investigated dupilumab, an anti-IL-4R mAb, 1 investigated REGN1908-1909, an anti-Fel d 1 mAb, and 2 investigated REGN5713/14/15, an anti-Bet v 1 mAb. For the anti-IgE mAbs, 2 used rhuMAb-E25, the former name of omalizumab, 4 used omalizumab, and 1 used quilizumab. Additionally, the follow-up time ranged from 8 to 28 weeks, and detailed intervention strategies were shown in Table 1. Moreover, Table 2 summarized the baseline demographics of 2630 included patients, of which 1598 belonged to the treatment group. All included studies recruited adults and patients’ mean ages were greater than 30, while 4 studies also recruited patients younger than 18 years old. All the studies recruited both male and female patients. 7 studies reported the detailed race composition of included patients and 6 studies provided information on weight, either range or mean. Among all 12 studies, 7 of them recruited SAR patients, 4 recruited PAR patients, and 1 recruited both. The SAR patients were induced by ragweed, birch pollen, Japanese cedar pollen, and grass pollen and 1 PAR study chose cat-induced PAR patients. 7 studies examined total serum IgE and reported the mean. There were 8 studies that recorded the asthma history of patients, of which 1 study excluded patients with asthma history, and 2 studies included patients with comorbid asthma history. Only 4 studies reported patients’ history of atopic dermatitis. Further information on other comorbid atopic diseases were not available.

**Figure 2:**
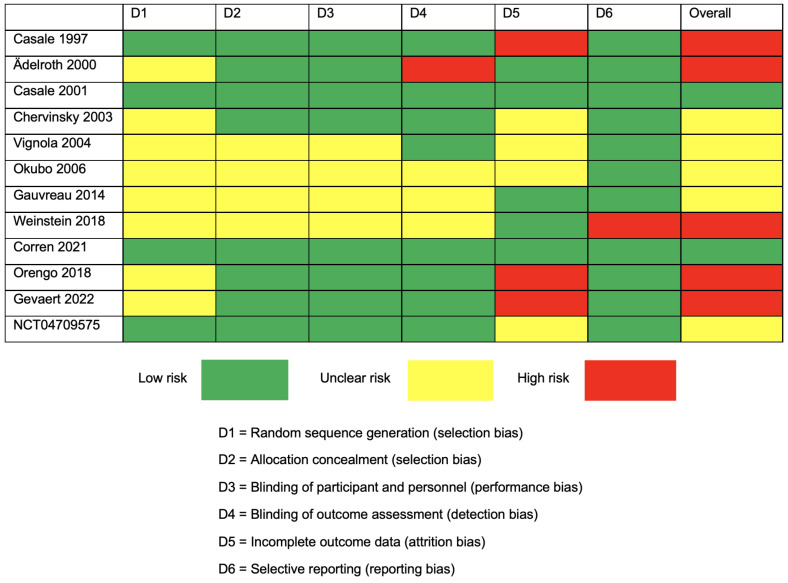
Traffic light plot for Cochrane Collaboration’s proposal for the assessment of the risk of bias (RoB 2) for RCTs [19,20,21,22,23,24,25,26,27,28,29,30].

The quality assessment results were presented using a traffic light plot in Figure 2. According to the assessment, 2 studies were at low risk, 5 studies were at unclear risk, and 5 studies had a high risk of bias. Most of the unclear risk came from the random sequence generation process, leading to a selection bias. The highest risk of bias among the different studies was noted in the domain of incomplete outcome data, causing an attrition bias.

### 3.2. Evidence for Adverse Events

Adverse events reported in all 12 including RCTs were summarized in Table 3. Among them, 10 studies provided data about total subjects experiencing adverse events, in the treatment and control group, respectively. All studies documented the frequently reported adverse events and their frequencies. Researchers individually evaluated the severity of adverse events as 3 levels: mild, moderate, and severe. “Severe” adverse events were defined to cause severe discomfort and limited normal function, leading to a significant change from baseline and definite damage to health, or probably required a prolonged hospitalization [19]. We specifically noted adverse events leading to withdrawal of the study and serious adverse events (as opposed to severe).

#### 3.2.1. Anti-IgE mAbs

Adverse events were reported in 6 omalizumab [19,20,21,22,23,24] and 1 quilizumab [25] trials. Except for 2 studies with no data reported, 373 out of 590 (63.2%) mAb-treated patients and 309 out of 489 (63.2%) placebo-treated patients experienced any adverse events during the trials. Frequently reported adverse events of omalizumab were infections, including upper respiratory infections, viral infections or influenza, nasopharyngitis, pharyngitis, and sinusitis; neurological symptoms, including headache, sinus headache, and special senses; respiratory symptoms, including coughing, worsen asthma, and sore throat; gastrointestinal symptoms, including nausea, colitis ulcerative, and diarrhea; musculoskeletal symptoms, including arthralgia, back pain, and sprains and strains; general and administration site reactions, including injection site reactions and urticaria; and systemic reactions, such as weight gain and skin and subcutaneous tissue disorders. The occurrence rates of severe, discontinuation-causing, and serious adverse events were 2.5%, 0.72%, and 8% for omalizumab-treated patients, and 5.4%, 0.49%, and 9.3% for placebo-treated patients, respectively. The study on quilizumab mainly reported infectious adverse events, including upper respiratory tract infections (29.2% in treatment group, and 16.7% in control group) and 1 severe gastroenteritis in the control group. 

#### 3.2.2. Anti-IL-4R mAb

For 2 included dupilumab trials, a total of 292 patients were included [26,27]. 77.6% dupilumab-treated patients and 74.3% placebo-treated patients experienced adverse events. Both studies reported injection site reactions, and 1 study also reported respiratory tract infections, neurological symptoms, and nasal congestions. There were no substantial differences between the 2 groups for severe and serious adverse events. However, patients in the treatment group had a higher possibility to discontinue the study due to adverse events than the control group (4.37% and 0.92%, respectively).

#### 3.2.3. Anti-Fel d 1 mAb

There was only 1 trial reporting adverse events comparing the safety of REGN1908-1909 treatment and placebo [28]. The occurrence rates of adverse events were similar in 2 groups (63.9% REGN1908-1909-treated patients and 62.2% placebo-treated patients), and none of them withdrew the trial because of adverse events. Frequently reported adverse events included infections, neurological symptoms, gastrointestinal symptoms, and injection site reactions, and each group reported 1 serious adverse event.

#### 3.2.4. Anti-Bet v 1 mAb

Two studies investigated the safety of REGN5713/14/15 [29,30], and a total of 413 patients were recruited. Overall, 118 out of 205 (57.6%) REGN5713/14/15-treated patients and 115 out of 208 (55.3%) placebo-treated patients suffered from at least 1 adverse event, showing no significant difference. Among them, 1 patient in the treatment group withdrew from the study, and 3 serious adverse events happened in the treatment group, with an occurrence rate of 0.73%. Frequently reported adverse events included headache, nasopharyngitis, and vaccination complication such as injection site reactions and hypersensitivity. 

### 3.3. Meta-Analysis Results 

**Figure 3:**
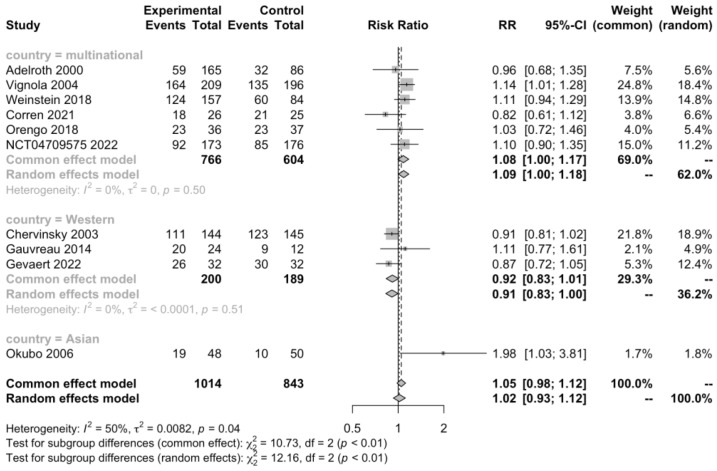
Forest plot of RCTs comparing patients experiencing adverse events subgrouped by country [20,22,23,24,25,26,27,28,29,30].

Ten RCTs (n = 1857) informed the total subjects experiencing adverse events, and the test for heterogeneity indicated a medium heterogeneity (I^2^ = 49.7%, *p* = 0.0365). Subgroup analysis was conducted to detect the source of heterogeneity, and we found that country played a significant role (I^2^ = 0%, *p* = 0.5, Figure 3), but subgrouping based on type of AR, age group of patients, and type of mAb didn’t reduce heterogeneity (Figures S1–S3). However, neither the subgroup analysis nor the overall random effects model (RR 1.02, 95% CI 0.93–1.12, Figure 3) showed significant differences in experiencing adverse reactions between the mAb-treatment and placebo group. There was no strong evidence of publication bias.

**Figure 4:**
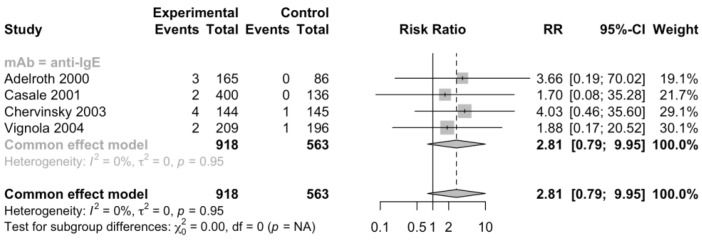
Forest plot of RCTs comparing rates of patients with urticaria subgrouped by type of mAb [20,21,22,23].

Regarding common adverse events, the risk of experiencing headache (RR 0.88, 95% CI 0.73–1.06, Figure S4), upper respiratory infection (RR 1.05, 95% CI 0.76–1.47, Figure S5), viral infection (RR 0.9, 95% CI 0.59–1.36, Figure S6), pharyngitis (RR 1.12, 95% CI 0.69–1.81, Figure S7), nasopharyngitis (RR 0.92, 95% CI 0.72–1.17, Figure S8), injection site reactions (RR 1.21, 95% CI 0.85–1.73, Figure S9), and urticaria (RR 2.81, 95% CI 0.79–9.95, Figure 4) were not significantly different between patients treated with monoclonal antibodies and placebo. Among them, pharyngitis and urticaria occurred only in omalizumab trials, and it was reasonable to speculate that patients treated with omalizumab might have an increased risk to experience urticaria though it failed to reach statistically significant. There was no strong evidence of heterogeneity.

**Figure 5:**
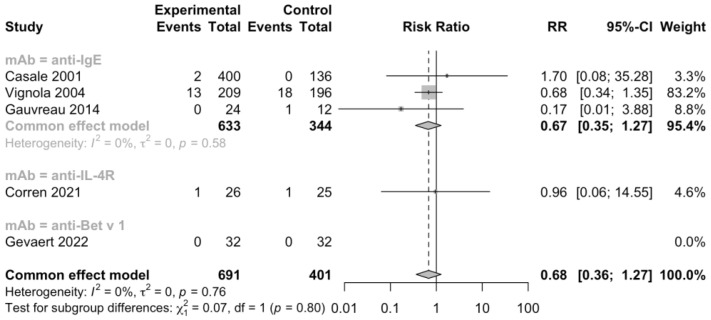
Forest plot of RCTs comparing severe adverse events subgrouped by type of mAb [21,23,25,27,29].

**Figure 6:**
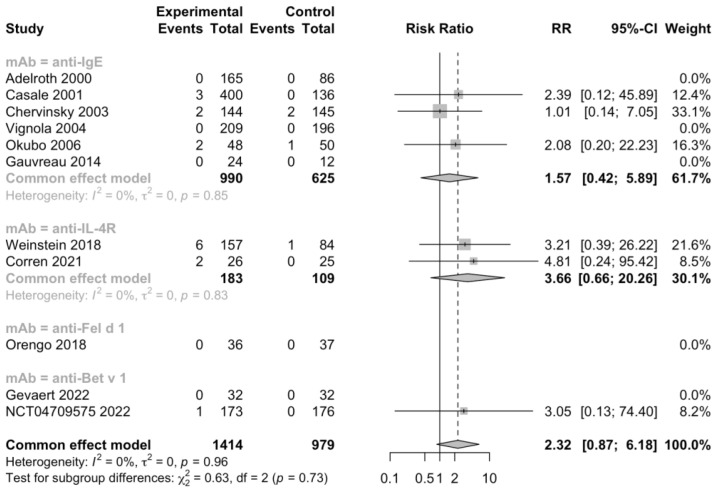
Forest plot of RCTs comparing withdrawal-causing adverse events subgrouped by type of mAb [20,21,22,23,24,25,26,27,28,29,30].

**Figure 7:**
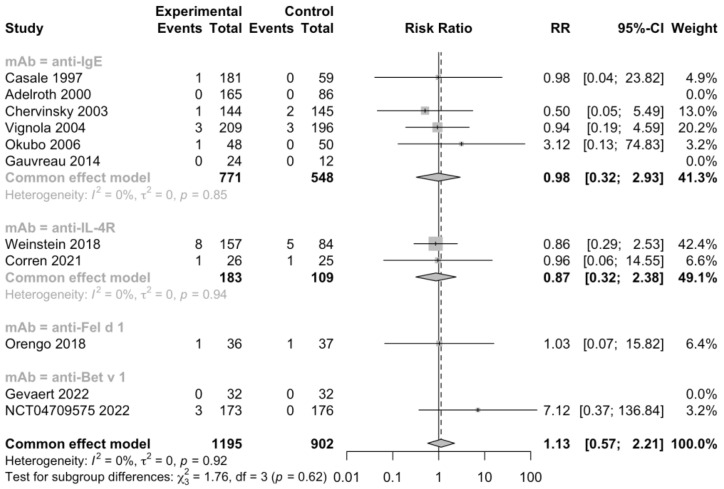
Forest plot of RCTs comparing serious adverse events subgrouped by type of mAb [19,20,22,23,24,25,26,27,28,29,30].

Additionally, 5 RCTs (n = 1092) informed severe adverse events, 11 RCTs (n = 2393) informed adverse events leading to discontinuation, and 11 RCTs (n = 2097) informed serious adverse events (Figure 5, Figure 6 and Figure 7). No heterogeneity was detected. The risk ratio of severe adverse events was 0.68, with a 95% CI of 0.36–1.27, indicating that treatment with mAbs reduced 38% risk of severe adverse events. In contrast, the risk ratio of adverse events leading to discontinuation was 2.32 and the 95% CI was 0.87–6.18, which showed that patients treated with mAbs might have a higher possibility to discontinue due to adverse events. However, both the severe and withdrawal-causing adverse events failed to reach statistically significant, and there was also no significant difference in the risk of serious adverse events (RR 1.13, 95% CI 0.57–2.21).

## 4. Discussion

This systematic review and meta-analysis of more than 2600 patients with AR in 12 RCTs assessed the safety of 5 monoclonal antibodies, including 2 anti-IgE mAbs omalizumab and quilizumab, 1 Anti-IL-4R mAb dupilumab, 1 Anti-Fel d 1 mAb REGN1908-1909, and 1 Anti-Bet v 1 mAb REGN5713/14/15, and provided comprehensive evidence on comparing the incidence of adverse events of treatment with mAbs and placebo. According to the pathogenesis of AR, type 2 immunity plays an important role [4]. Thus, type 2 cytokines, such as IL-4, IL-5, and IL-13, are promising therapeutical targets for AR and also other allergic diseases, such as asthma and atopic dermatitis [7,9]. Unfortunately, according to our literature review, there were currently no RCTs investigating the safety of anti-IL-5/5Ra mAbs including mepolizumab, reslizumab and benralizumab, or anti-IL-13 mAbs including lebrikizumab and tralokinumab in AR. Further research on the tolerability of other types of mAbs should be conducted before more biological therapies are introduced to AR patients.

Our results demonstrated that total, serious, and common adverse events such as headache, upper respiratory infection, viral infection, pharyngitis, nasopharyngitis, and injection site reactions occurred with a similar incidence rate in the mAb and placebo groups, indicating the acceptable safety and tolerability of biologics in patients with AR in general. Our result was similar to the conclusions of other meta-analysis researches on the safety of biological treatments in AR, especially omalizumab [8,31]. We also found that countries where studies were conducted brought up a medium heterogeneity, and subgrouping the studies into Western, Asian, and multinational could eliminate heterogeneity, which indicated that the region of research might influence the incidence of adverse events. Region could not only reflect the racial compositions of participants to some extent, but also give a hint of the living environments and lifestyles of patients, which was proved to be essential factors causing AR, and even many other allergic diseases [32,33,34,35]. As AR has become a global health issue, the promotion of biological treatments worldwide required special attention on adverse events among patients in different areas with different ethnicities. According to ICH E5, frameworks were needed to register and review the global trials and decide the acceptability of foreign clinical data across different races [36]. Some studies found that the efficacy and safety of biological treatments varied in different populations. For example, a cohort study showed different rates of response and adverse effects to biological therapies between South Asian IBD patients, mainly migrants, and Caucasian American [37]. However, there was currently no information comparing tolerability differences of AR patients across regions. Therefore, future studies must address this issue to guide the clinical use of monoclonal antibodies in AR patients from different countries. 

Furthermore, we suspected urticaria to be an adverse event at high risk for treatment with omalizumab, although the data failed to reach statistical significance. Systemic hypersensitivity reactions was found to be associated with multiple biological treatments such as omalizumab, dupilumab, and benralizumab [38,39]. In spite of some evidence showing that hypersensitivity reactions including urticaria, skin rush, and anaphylaxis had a low incidence rate and no significant difference between omalizumab and placebo-treated patients [40], anaphylaxis has been proved to be related to omalizumab since its initial FDA approval for allergic asthma in 2003 and the product label even includes a black box warning [41,42]. In the future, more investigations should evaluate the hypersensitive adverse reactions of biological therapies on allergic patients, especially AR. For novel monoclonal antibodies targeting allergic proteins, such as REGN1908-1909 which targets Fel d 1, the dominant allergen in cat dander triggering AR and asthma symptoms [43], and REGN5713/14/15 which targets Bet v 1, the most abundant and immunodominant allergen in birch pollen triggering birch-related AR^12^, the overall safety was acceptable based on the analysis of the included studies. However, the number of trials and patients included were insufficient, and there was no past research indicating tolerability information in other diseases for reference. Thus, more studies are expected to draw a strong conclusion on the safety of innovatory biologics for the advancement of future clinical drugs.

The strengths of our study included a comprehensive and up-to-date search with restricted selection criteria following scientific guidelines. All the included studies were placebo controlled RCTs, and quality assessment demonstrated that most studies were at low or unclear risk of bias. Additionally, there was no evidence of statistical heterogeneity in all the meta-analysis outcomes (one eliminated heterogeneity after subgrouping), strengthening the consistency and reliability of our analysis. However, several limitations of the evidence should be considered. The number of studies in quilizumab, dupilumab, REGN1908-1909, and REGN5713/14/15 was small (≤2 studies for each biological product), providing limited safety information, especially for adverse events with extremely low incidence rates. Besides, all the studies had a relatively short follow-up time (the maximum follow-up time was 28 weeks), and this might lead to the ignorance of some adverse events in long-term practical use. Finally, some studies reported the safety data incompletely. For example, they only reported frequent adverse events with an incidence rate >10% and failed to summarize the severity of adverse events.

In conclusion, this systematic review with meta-analysis indicated that biological therapies were well tolerated in patients with AR, with no common or serious adverse events reaching statistical significance. Country was a significant factor for heterogeneity, and hypersensitivity such as urticaria required special caution. From a safety perspective, monoclonal antibodies are promising novel treatment options for different types of AR. We expect more investigations and future studies to confirm our findings and assess the long-term safety of biologics in the use of AR patients worldwide.

## Figures and Tables

**Table 1:** Characteristics of included randomized controlled trials (RCTs).

Study	Country	Sample Size	Experimental Sample Size	Control Sample Size	Monoclonal Antibody	Intervention	Follow-Up Time
Anti-IgE							
Casale 1997 [19]	USA	240	181	59	rhuMAb-E25 (Omalizumab)	Initial IV loading dose on day 0 + 0.15 mg/kg SC/IV, 0.5 mg/kg IV or placebo SC/IV on days 7, 14, 28, 42, 56, 70, 84	8 weeks
Ädelroth 2000 [20]	Sweden, Finland, and Norway	251	165	86	rhuMAb-E25 (Omalizumab)	300 mg SC or placebo at weeks 0 and 4 for baseline serum IgE ≤ 150 IU/mL or at weeks 0, 3 and 6 for baseline serum IgE > 150 IU/mL	12 weeks
Casale 2001 [21]	USA	536	400	136	Omalizumab	50, 150 or 300 mg SC or placebo at weeks 0, 3, 6, 9 for baseline serum IgE ≤ 150 IU/mL or at weeks 0, 4, 8 for baseline serum IgE > 150 IU/mL	24 weeks
Chervinsky 2003 [22]	USA	289	144	145	Omalizumab	At least 0.016 mg/kg/IgE [IU/mL] SC or placebo q2/4w based on baseline serum free IgE and body weight for 16 weeks	NR
Vignola 2004 [23]	Argentina, Belgium, Canada, Denmark, Finland, France, Germany, Italy, Norway, Sweden, and UK	405	209	196	Omalizumab	At least 0.016 mg/kg/IgE [IU/mL] or placebo q2/4w based on baseline serum free IgE and body weight for 28 weeks	NR
Okubo 2006 [24]	Japan	98	48	50	Omalizumab	150, 225, 300, or 375 mg SC or placebo q2/4w based on baseline serum total IgE and body weight for 12 weeks	12 weeks
Gauvreau 2014 [25]	Canada	36	24	12	Quilizumab	1.5 or 5.0 mg/kg IV or 3.0 mg/kg SC or placebo q28d for 3 months	16 weeks
Anti-IL-4R							
Weinstein 2018 [26]	Argentina, Australia, Chile, France, Italy, Japan, Korea, Mexico, New Zealand, Poland, Russia, South Africa, Spain, Turkey, Ukraine, and USA	241	157	84	Dupilumab	200 or 300 mg SC or placebo q2w for 24 weeks	16 weeks
Corren 2021 [27] *	Canada and USA	51	26	25	Dupilumab	600 mg loading dose on Day 1 + 300 mg SC q2w or placebo for 16 weeks	8 weeks
Anti-Fel d 1							
Orengo 2018 [28]	Netherlands, New Zealand, Sweden, and UK	73	36	37	REGN1908-1909	A single dose SC (600 mg total, 1:1 antibody ratio) or placebo on day 1	85 days
Anti-Bet v 1							
Gevaert 2022 [29]	Belgium	64	32	32	REGN5713/14/15	A single 900 mg dose SC (300 mg/antibody) or placebo	16 weeks
NCT04709575 [30]	Belgium, Canada, Denmark, Germany, and USA	353	176	177	REGN5713/14/15	A single 900 mg dose of REGN5713-5714-5715 or placebo	28 weeks

NR: data not reported; * Only patients in placebo and dupilumab groups were included in this review; IV: intravenous; SC: subcutaneous; q2w: every 2 weeks; q4w: every 4 weeks; q28d: every 28 days.

**Table 2:** Baseline demographics of included patients.

Study	Age Range, Years (Mean Age)	Sex, n (%)		Race, n (%)				Weight Range, kg (Mean Weight)	Type of AR	Mean IgE, IU/mL	History of Asthma, n (%)	History of Atopic Dermatitis, n (%)
		Male Female		White Black Asian Others								
Anti-IgE												
Casale 1997 [19]	18–66 (34)	116 (48)	124 (52)	209 (87)	22 (9)	0	9 (4)	44–153 (77)	SAR (ragweed-induced)	240	33 (13.8)	NR
Ädelroth 2000 [20]	17–66 (33)	117 (47)	134 (53)	246 (98)	0	0	5 (2)	48–110 (73.3)	SAR (birch pollen-induced)	144	85 (34)	49 (19.5)
Casale 2001 [21] *	12–75 (34.5)	240 (45)	289 (55)	NR (92–96)	NR	NR	NR	NR (72.8)	SAR (ragweed-induced)	173.6	137 (26)	NR (12–15)
Chervinsky 2003 [22]	12–75 (34.5)	112 (39)	177 (61)	NR				≤150 (NR)	PAR (dust mite, dog or cat-induced)	147.5	76 (26)	49 (17)
Vignola 2004 [23]	12–74 (38.4)	182 (45)	223 (55)	NR				NR	PAR (indoor allergen-induced)	NR	405 (100)	NR
Okubo 2006 [24]	20–64 (31.8)	53 (54)	45 (46)	NR				30–150 (NR)	SAR (Japanese cedar pollen-induced)	191	NR	NR
Gauvreau 2014 [25]	NR (35)	17 (47)	19 (53)	35 (97)	1 (2.8)	0	0	NR (72)	PAR or SAR	167.7	0	NR
Anti-IL-4R												
Weinstein 2018 [26]	≥18 (46.5)	92 (38)	149 (62)	171 (71)	19 (8)	45 (19)	6 (2)	NR	PAR (Aspergillus fumigatus, cat, dust mite, dog, German cockroach, or Oriental cockroach-induced)	NR	241 (100)	26 (10.8)
Corren 2021 [27] *	18–55 (37.5)	25 (49)	26 (51)	NR				NR	SAR (grass pollen-induced)	159.8	16 (31.4)	NR
Anti-Fel d 1												
Orengo 2018 [28]	18–55 (27.8)	36 (49)	37 (51)	62 (85)	0	7 (10)	4 (5)	NR	PAR (cat-induced)	NR	NR	NR
Anti-Bet v 1												
Gevaert 2022 [29]	18–60 (36.9)	22 (34)	42 (66)	63 (98.4)	0	0	1 (1.6)	NR	SAR (birch pollen-induced)	NR	NR	NR
NCT04709575 [30]	≥18 (40.9)	149 (42.2)	204 (57.8)	326 (92.4)	3 (0.8)	13 (3.7)	11 (3.1)	NR	SAR (birch pollen-induced)	NR	NR	NR

NR: data not reported; * 7 patients with no data within the pollen season were excluded from the demographic summary; SAR: seasonal allergic rhinitis; PAR: perennial allergic rhinitis.

**Table 3:** Adverse events among the included studies.

Study	Total Subjects Experiencing Adverse Events		Frequently Reported Adverse Events *		Severe Adverse Events		Adverse Events Leading to Withdrawal		Serious Adverse Events	
	TRG (%)	CRG (%)	TRG	CRG	TRG(#)	CRG(#)	TRG(#)	CRG(#)	TRG(#)	CRG(#)
Anti-IgE										
Casale 1997 [19]	NR	NR	Headache, infection, pharyngitis, pain	Headache, infection, pain, pharyngitis	NR	NR	NR	NR	Colitis (1)	0
Ädelroth 2000 [20]	59 (36)	32 (37)	Injection site reactions, localized urticaria	Injection site reactions	NR	NR	0	0	0	0
Casale 2001 [21]	NR	NR	Headache, upper respiratory infection, viral infection, pharyngitis, sinusitis, weight gain, sinus headache, back pain, arthralgia, coughing, sprains and strains, nausea, special senses, worsen asthma, urticaria	Headache, sinusitis, upper respiratory infection, viral infection, back pain, special senses, worsen asthma, arthralgia, coughing, sprains and strains, weight gain, pharyngitis, sinus headache	sprains and strains (1), nausea (1)	0	3	0	NR	NR
Chervinsky 2003 [22]	111 (77.1)	123 (84.8)	Upper respiratory infection, headache, nasopharyngitis, sore throat, sinusitis, influenza, back pain, sinus headache, urticaria, injection site reactions	Headache, nasopharyngitis, upper respiratory infection, sinusitis, influenza, sinus headache, back pain, sore throat, injection site reactions, urticaria	NR	NR	Headache and nasopharyngitis (1), urticaria (1)	Headache (1), syncope (1)	Infectious mononucleosis (1)	Appendicitis (1), recurrent disk herniation (1)
Vignola 2004 [23]	164 (78.5)	135 (68.9)	Nasopharyngitis, headache, general and administration site reactions, injection site reactions, influenza, sinusitis, pharyngitis, upper respiratory infection, urticaria	Nasopharyngitis, headache, pharyngitis, influenza, upper respiratory infection, injection site reactions, general and administration site reactions, sinusitis, urticaria	13	18	0	0	Acute appendicitis (1), mild chest pain (1), mild depression (1)	Intestinal obstruction (1), atrial fibrillation (1), serious asthma exacerbation (1)
Okubo 2006 [24]	19 (39.6)	10 (20)	General and administration site reactions, investigations, colitis ulcerative, skin and subcutaneous tissue disorders	General and administration site reactions, investigations, headache, skin and subcutaneous tissue disorders, diarrhea	NR	NR	2	1	colitis ulcerative (1)	0
Gauvreau 2014 [25]	20 (83)	9 (75)	Upper respiratory tract infection	Upper respiratory tract infection	0	Gastroenteritis (1)	0	0	0	0
Anti-IL-4R										
Weinstein 2018 [26]	124 (79)	60 (71.4)	Injection site reactions	Injection site reactions	NR	NR	6	1	8	5
Corren 2021 [27]	18 (69.2)	21 (84)	Injection site reactions, nasopharyngitis, upper respiratory tract infection, headache, nasal congestion	Injection site reactions, nasopharyngitis, upper respiratory tract infection, headache, nausea, viral upper respiratory tract infection, nasal congestion	1	1	2	0	1	1
Anti-Fel d 1										
Orengo 2018 [28]	23 (63.9)	23 (62.2)	Headache, upper respiratory tract infection, cough, nausea, rhinitis, abdominal pain, dizziness, gastroenteritis, viral upper respiratory tract infection, vomiting, nasopharyngitis, injection site reactions	Headache, upper respiratory tract infection, nasopharyngitis, abdominal pain, eczema, nasal congestion, viral upper respiratory tract infection, cough, dizziness, injection site reactions, nausea, rhinitis, vomiting	NR	NR	0	0	Pyelonephritis (1)	Appendicitis (1)
Anti-Bet v 1										
Gevaert 2022 [29]	26 (81.3)	30 (93.8)	Headache, injection site reactions, nasopharyngitis, hypersensitivity, sinusitis	Nasopharyngitis, headache, injection site reactions, sinusitis, hypersensitivity	0	0	0	0	0	0
NCT04709575 [30] **	92 (53)	85 (48)	Vaccination complication, headache, nasopharyngitis	Vaccination complication, headache, nasopharyngitis	NR	NR	1	0	Appendicitis (1), thermal burn (1), breast cancer (1)	0

TRG: treatment group; CRG: control group; NR: data not reported; * Adverse events were sorted by frequency, and then alphabetical order; #: number of patients; ** Only 173 patients in the treatment group and 176 patients in the control group were analyzed.

## Data Availability

Not applicable.

## Supplementary Materials

The following supporting information can be downloaded at: https://www.mdpi.com/article/10.3390/jcm12082848/s1, Figure S1: Forest plot of RCTs comparing patients experiencing adverse events subgrouped by AR type [20,22,23,24,25,26,27,28,29,30]; Figure S2. Forest plot of RCTs comparing patients experiencing adverse events subgrouped by age group [20,22,23,24,25,26,27,28,29,30]; Figure S3. Forest plot of RCTs comparing patients experiencing adverse events subgrouped by type of mAb [20,22,23,24,25,26,27,28,29,30]; Figure S4. Forest plot of RCTs comparing rates of patients with headache subgrouped by type of mAb [19,21,22,23,24,27,28,29,30]; Figure S5. Forest plot of RCTs comparing rates of patients with upper respiratory infection subgrouped by type of mAb [21,22,23,25,27,28]; Figure S6. Forest plot of RCTs comparing rates of patients with viral infection subgrouped by type of mAb [21,22,23,27,28]; Figure S7. Forest plot of RCTs comparing rates of patients with pharyngitis subgrouped by type of mAb [19,21,23]; Figure S8. Forest plot of RCTs comparing rates of patients with nasopharyngitis subgrouped by type of mAb [22,23,27,28,29,30]; Figure S9. Forest plot of RCTs comparing rates of patients with injection site reactions subgrouped by type of mAb [20,22,23,26,27,28,29].
