# Supplementary material for: Adverse Events for Monoclonal Antibodies in Patients with Allergic Rhinitis: A Systematic Review and Meta-Analysis of Randomized Clinical Trials
Source: J Clin Med. 2023 Apr 13;12(8):2848. doi: 10.3390/jcm12082848 (PMC10144224; doi:10.3390/jcm12082848)
Supplement: Supplementary file 1 [file jcm-12-02848-s001.zip › jcm-2218962-supplementary.pdf]

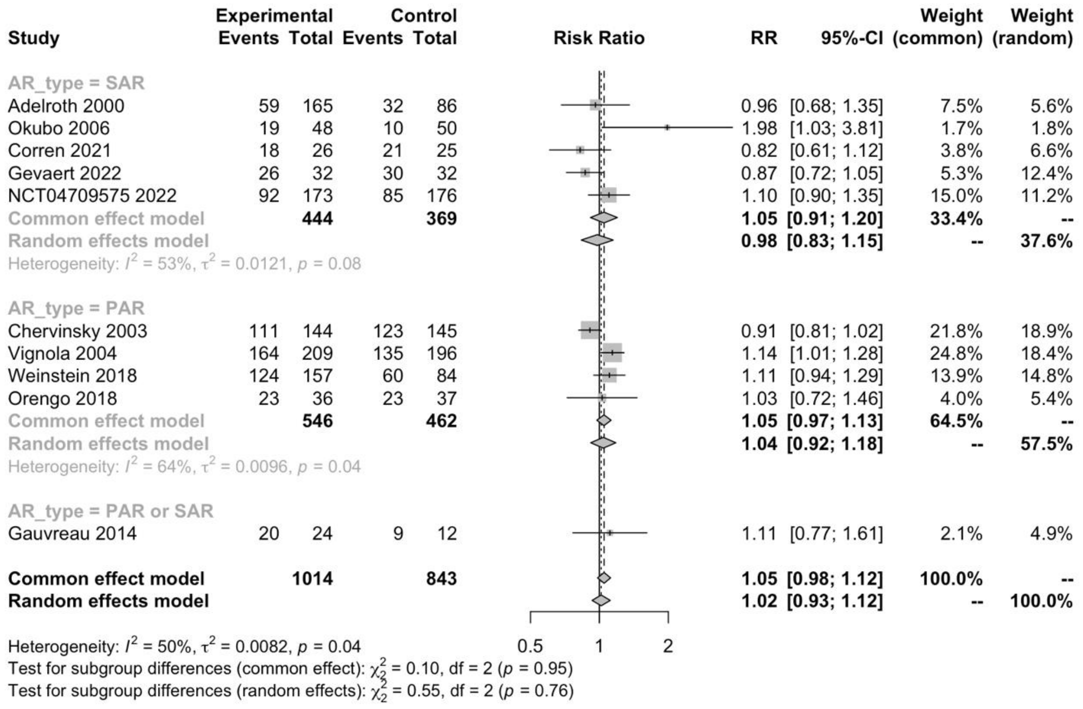

Figure S1. Forest plot of RCTs comparing patients experiencing adverse events subgrouped by AR type [20,22–30].

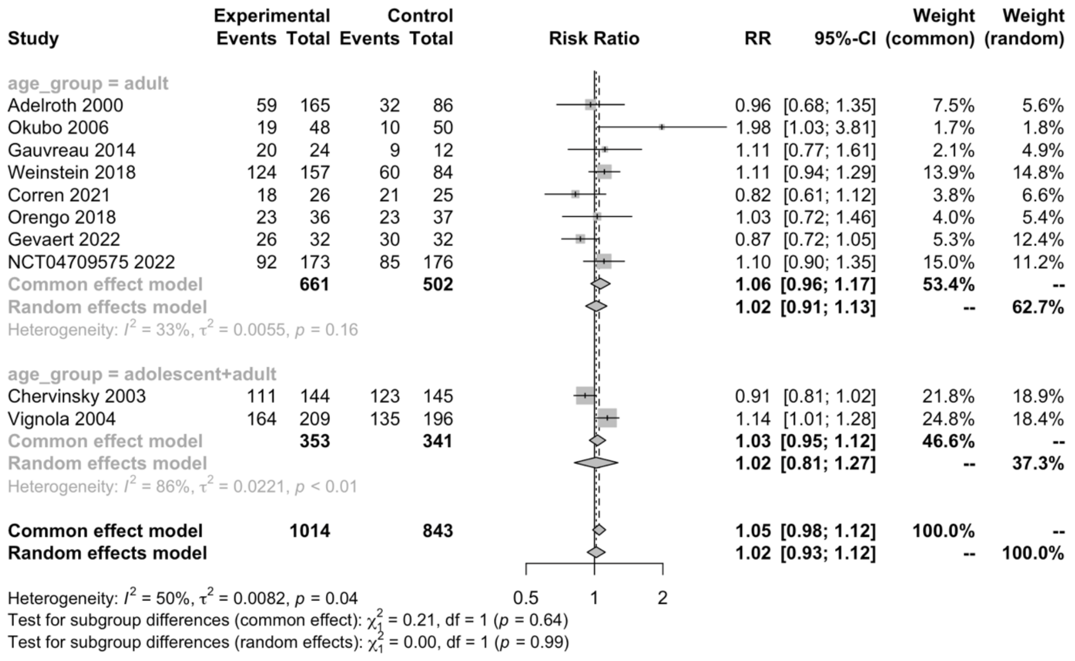

Figure S2. Forest plot of RCTs comparing patients experiencing adverse events subgrouped by age group [20,22–30].

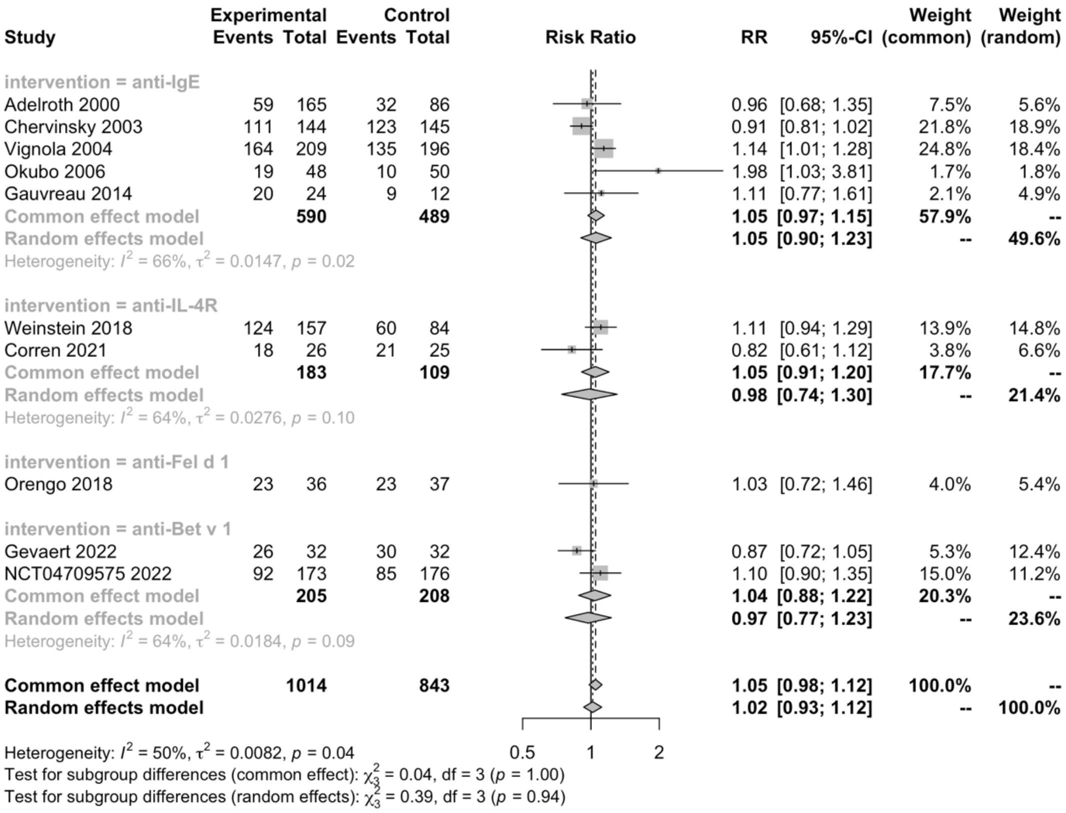

Figure S3. Forest plot of RCTs comparing patients experiencing adverse events subgrouped by type of mAb [20,22–30].

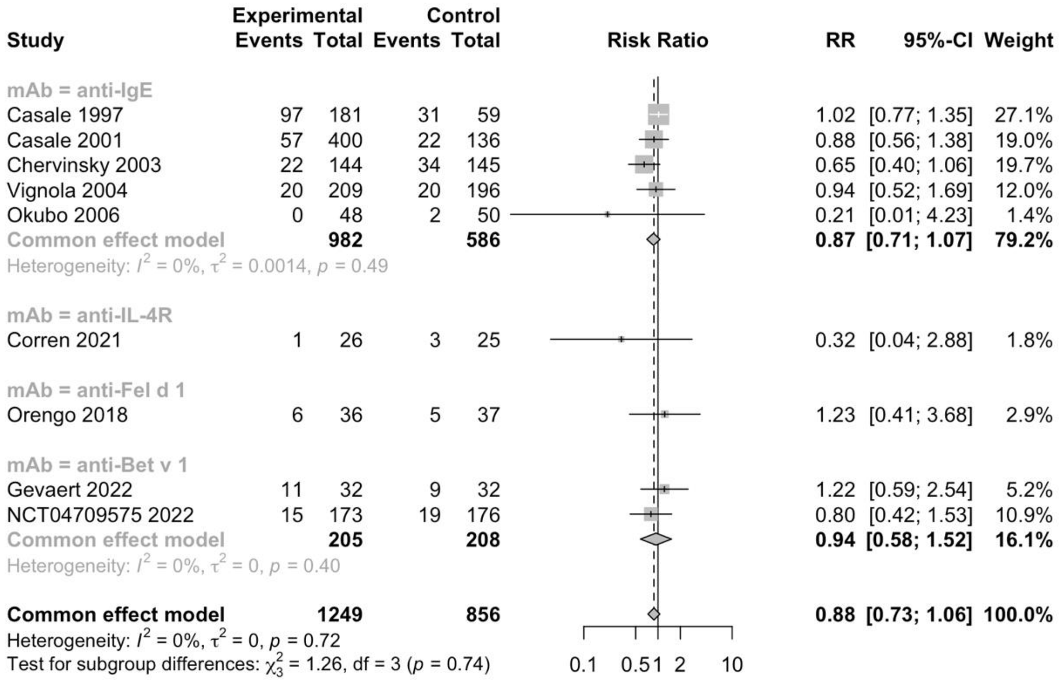

Figure S4. Forest plot of RCTs comparing rates of patients with headache subgrouped by type of mAb [19,21–24,27–30].

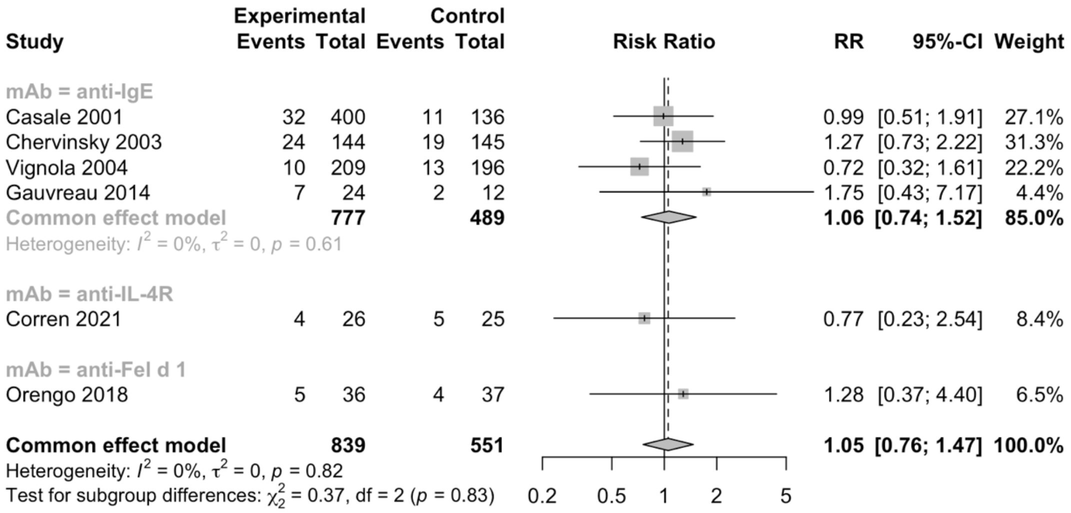

Figure S5. Forest plot of RCTs comparing rates of patients with upper respiratory infection subgrouped by type of mAb [21–23,25,27,28].

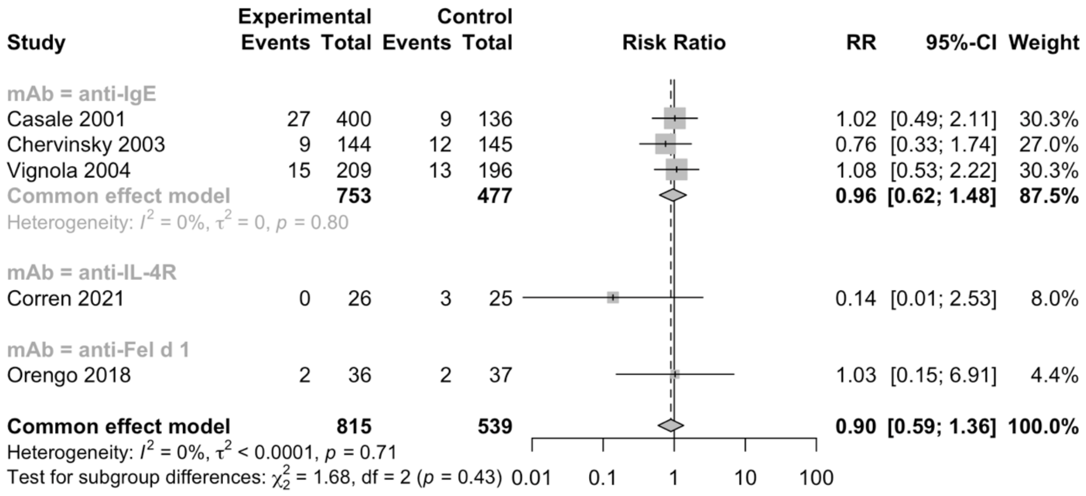

Figure S6. Forest plot of RCTs comparing rates of patients with viral infection subgrouped by type of mAb [21–23,27,28].

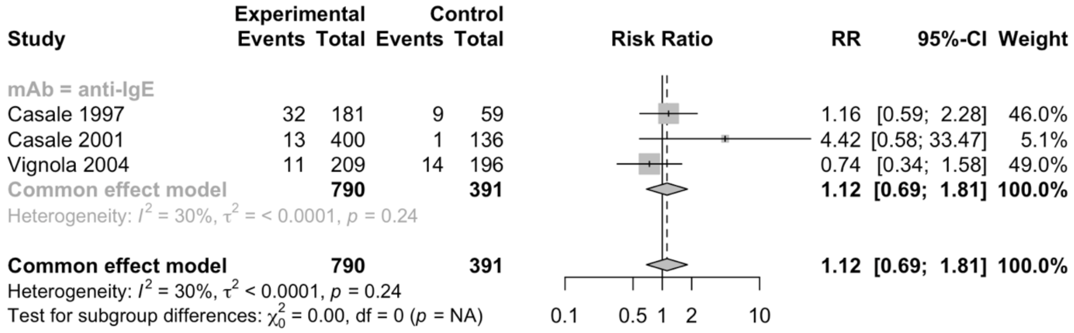

Figure S7. Forest plot of RCTs comparing rates of patients with pharyngitis subgrouped by type of mAb [19,21,23].

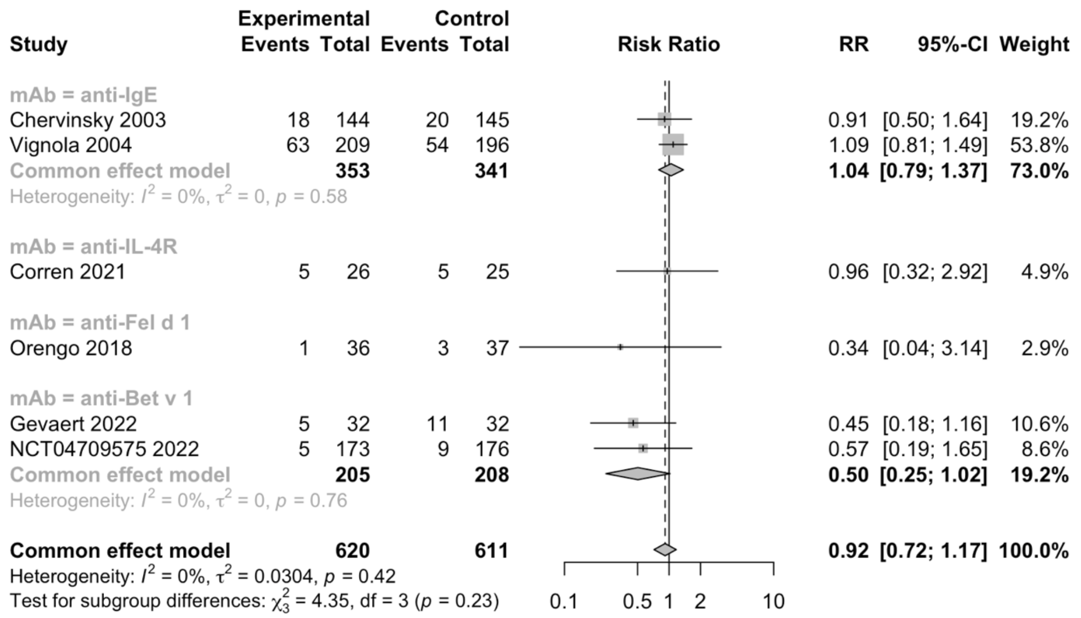

Figure S8. Forest plot of RCTs comparing rates of patients with nasopharyngitis subgrouped by type of mAb [22,23,27–30].

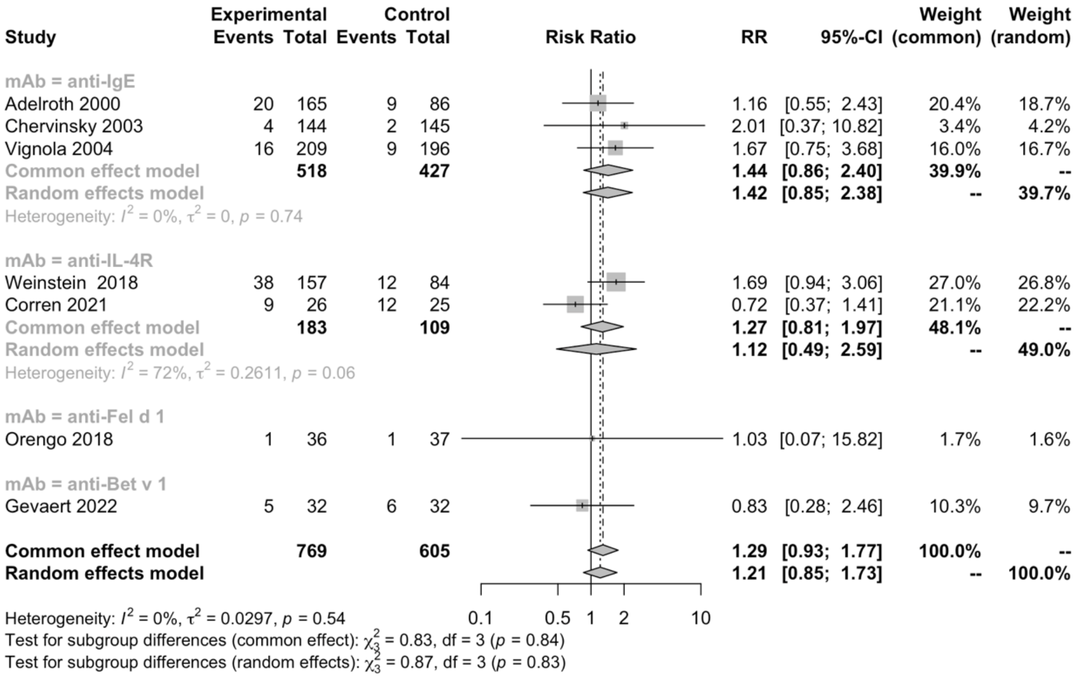

Figure S9. Forest plot of RCTs comparing rates of patients with injection site reactions subgrouped by type of mAb [20,22,23,26–29].
